# Dual Role of Bacteria in Carcinoma: Stimulation and Inhibition

**DOI:** 10.1155/2020/4639761

**Published:** 2020-08-24

**Authors:** Suad A Al-Hilu, Wisam H Al-Shujairi

**Affiliations:** ^1^Department of Biology/Faculty of Sciences, University of Kufa, 54001 Najaf, Iraq; ^2^Department of Clinical Laboratory Sciences/College of Pharmacy, University of Babylon, 51001 Hilla, Iraq

## Abstract

Although what unifies the carcinogenic microorganisms has not been determined by multiple studies, the role of bacteria in the development of neoplasms has not been properly elucidated. In this review, we discuss links between the bacterial species and cancer, with focus on immune responses for the stimulation of tumor cells such as induction of inflammation. Finally, we will describe the potential therapeutic strategies of bacteria on target tumors to improve treatment while mitigating adverse reactions. Cancer is a series of genetic changes that transform normal cells into tumor cells. These changes come from several reasons, including smoking, drinking alcohol, sunlight, exposure to chemical or physical factors, and finally chronic infection with microorganisms, including bacteria. In fact, bacterial infections are not carcinogenic, but recently it was discovered that the association between bacteria and cancer is through two mechanisms, the first stimulating chronic inflammation and the second producing carcinogenic metabolites. While bacteria are carcinogenic agents also, they have a dual role eliminating and removing tumor cells. However, the traditional cancer treatments that include chemotherapy, radiotherapy, surgery, and immunotherapy increase the chances of survival, and there are many side effects of these therapies, including the high toxicity of tissues and normal cells, could not penetrate the tumor cells, and resistance of these therapies by tumor cells. Therefore, the world has turned to an alternative solution, which is the use of genetically engineered microorganisms; thus, the use of living bacteria targeting cancerous cells is the unique option to overcome these challenges. Bacterial therapies, whether used alone or combination with chemotherapy, give a positive effect to treat multiple conditions of cancer. Also, bacteria can be used as vectors for drug, gene, or therapy, and this is a great step to treat cancer. Thus, we review the mechanisms underlying the interaction of the microbiota residents with cancer. Cancer-associated bacteria differ from those in healthy human and are linked with gene-expression profile. We also discuss how live bacteria interact with tumor microenvironments to induce tumor regression through colonization and spread. Finally, we provide past and ongoing clinical trials that include bacteria targeting tumors.

## 1. Introduction

Cancer remains to be one of the highest causes of morbidity and mortality throughout the world; it arises from the growth of malignant cells into masses referred to as tumors; they cause DNA mutations leading to acquisition of epigenetic changes promoting oncogenesis and carcinogenesis with several diseases [1]. Some of these cancers are spreading from their tissue to other parts in the body in a process called metastasis [2, 3]. Cancers can be categorized according to their tissue and/or organ of origin; carcinomas, for example, are cancers spreading in tissues covering all the body organs. Not all cancers are malignant; some cancers such as leukemia, lymphoma, and myeloma are referred to as nonsolid cancers [3]. Cancer causes series of genetic alterations elevated from several external factors including alcohol, smoking [4], and sunlight. Most of cancers in head and neck are caused by alcohol, and about 86% of skin cancer is raised from sunlight exposure [5, 6]. The death cases of cancer were recorded in 2018 to be about 18.1 million occurring in Asia, Europe, and Americas and will reach ~ 17 million deaths in 2030 [7]. The most common types cancer among men are lung, stomach, prostate, and colorectal cancer, while among women are lung, breast, cervix, and colorectal cancers [8]. Cell transformation is a process by which the normal cells altered to cancer cells; according to this belief, the studies around this field is very extensive but the mechanism of cancer remains unclear [9]. This information made it impossible to understand the progression of the tumor by which the bacteria may be the cause, or colonize, or treat the cancer [10]. Bacterial chronic infections are important cancer-related factors due to the effect of bacteria on cell cycle and its ability to attack the immune system and cause immune suppression [11] by several mechanisms including inflammation, lymphoproliferation, and induction of the hormones that increase the epithelial cell proliferation [12]. During inflammation, the phagocytes are recruited to the site of infection accompanied by secretion proinflammatory cytokines, such as tumor necrosis factor and chemokines which attracts other cells to the site of infection, which amplifies the immune response; thus, stimulation of renewed cell division occurs leading to mutations, deletions, or translocations as damaged DNA promotes the development of cancer cells [13]. The damage in DNA is similar to the carcinoma caused by the genes that altered the control of normal cells and finally apoptosis [10]. The germ theory of cancer was first proposed in the early 20^th^ century, and after that, they implicated the bacterium *Helicobacter pylori* and later *Fusobacterium nucleatum* in the development of gastric and colorectal cancers [14]. Several studies indicates that patients with colorectal cancer carry a large proportion of bacteria responsible for inflammatory diseases in the gastrointestinal tract, which is also able to produce toxins and oncogenic metabolites [15].

On the other hand, cancer therapies including chemotherapy, surgery, and radiotherapy as conventional therapies have increased rates of survival against cancer worldwide [16]; cancer treated by therapeutic strategies is called chemotherapy; it is employed worldwide, but most of these drugs are incapable of completely penetrating into the tumor location, because they are not specific and harm all the cells in the body (damaged and normal cells). Therefore, it is important to use other ways for treating cancer [17], because surgery removes the solid tumor and the use of continuous chemotherapy leads to resistance and finally an oxygen deficiency from the cancer environment that determines radiation penetration. All these reasons have made scientists think about using another alternative to treat cancer, and the best solution is bacteria,;they have the ability to kill cancer cells and the body does not have any resistance against them and remains sensitive in addition to the ability of bacteria to colonize in hypoxic core [18, 19]. For example, BCG for treatment of urinary bladder cancer was found very effective compared with chemotherapy, and trail use of *Salmonella enterica* in dogs affected with tumor reported benefit from the traditional treatment [20]. The effectiveness of cancer-targeting bacteria on tumor is not directly compared with that of the other cancer therapies, as the bacteria begin to affect cancer from the depth of the tumor, then followed by the antitumor immune response [21].

Bacteria cancer therapy (BCT) opens doors for cancer treatment completely, although the mechanism of bacteria to kill cancer cells is poorly understood [22]. The relationship between cancer and bacteria was first observed by two researchers Busch and Fehleisen; they showed that the patients with erysipelas infection caused by *Streptococcus* get carcinomas [23]. The microbiota (the commensal microorganisms in the human body) is necessary for healthy survival and regulation of function. On the other hand, the microbiota controls cancer during stages of predisposing conditions, initiation, susceptibility to immune response, genetic instability, progression, and interaction with the therapy [24]. The other therapeutic approaches of bacteria in cancer manipulation are DNA vaccine and antitumor metabolites [25].

However, since the interactions between the microbiome and the host are very diverse, it is difficult to determine their exact contributions to the development of cancer. In particular, it has been shown that pathogenic bacteria manipulate and exploit the position of the human host cell in different ways throughout different stages of the infection cycle. In this review, we highlighted on bacterial traits which make them carcinogenic agents and how live bacteria interact with tumor environment to stimulate tumor regression. And, we also provide examples of several bacterial species that induce the development of cancer. Finally, we provide different ways to engineer bacteria to improve effectiveness and safety for use as bacterial cancer therapies.

## 2. Cancer Induced by Bacterial Infection

**Figure 1:**
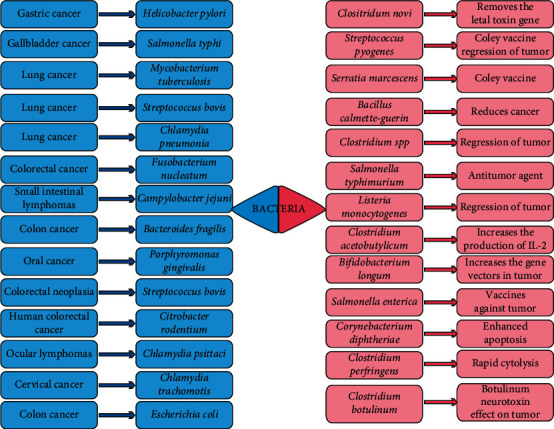
The dual role of bacteria that can be carcinogenic and treatable (updated from [34]).

Actually, the development and origin of tumor cells are unknown due to the transformations of cell need long time [9]. The cell transformation may occur spontaneously [26] by presence of carcinogenic physical factors such as X-ray induction [27], carcinogenic chemical factors [28], or microorganisms [29]. There is no evidence to prove the role of bacteria in the initiation of tumor, but microbiota may induce the further progress of cancer [30]. There are several studies that showed that the bacteria are carcinogenic and tumor-stimulating agents; they have the ability to produce toxins that change the cell regulating signals leading to cell growth regulation [31]. Normally, the immune system works against the tumor formation, but the chronic bacterial infection invades the immune system and induces the immune responses which play important roles in carcinogenesis by releasing cytokines from proinflammatory cells and free oxygen radicals such as reactive oxygen species (ROS) [32, 33]. Some strains of bacteria have been observed to be carcinogenic (Figure 1), for example, *Helicobacter pylori* with gastric cancer, *Salmonella typhi* with hepatobiliary carcinoma, *Campylobacter jejuni* with small intestinal lymphomas, *Chlamydia psittaci* with ocular lymphomas, *Mycobacterium tuberculosis* with lung cancer, and *Citrobacter rodentium* with human colorectal cancer [34]. Approximately 16% of all cancers in the world might be caused by microorganisms; especially liver cancer and gastrointestinal tract cancer were identified to be bacterial related [35]. Among these, colonic carcinoma was associated with the presence of endocarditis caused by *Streptococcus bovis* in 1951; in 1974, the association between *Streptococcus bovis* and colorectal neoplasia was recognized [36].

When it was discovered that bacteria are the cause of many infectious diseases, it was accepted that cancer does not act as an infectious or contagious disease. Thus, the concept of bacterial involvement was carcinogenic was unacceptable [37]. In 1890, Russel first introduced the possibility of cancer caused by bacteria. A few years later, Thomas Glove [38] in 1926 mentioned that certain bacteria could constantly be isolated from neoplastic tissue. In 1931, Hodgkin's acid-linked disease was found quickly to be caused by bacteria [39]. In 1931, it was observed that Hodgkin's disease was related with acid-fast bacteria [40]. Later, in 1941, George Mazet reported that both leukemia and Hodgkin's diseases were constantly linked to bacteria [41]. In 1953, White claimed that antibodies against anticancer bacteria had a protective effect. In 1953, Diller reported the isolation of highly polymorphic bacteria from the cancer tissue [42].

Besides the outer surface of bacteria is a complex structure which is capable of activating the immune system, the pathogenic bacteria have modifications in the outer surface for escape from the immune system to enhance the significant survival. For example, the outer surface of Gram-negative bacteria is covered with a polysaccharide capsule that limits the complement activation by structures like shield on the membranes of *E. coli*, *Haemophilus influenzae*, *Streptococcus pneumoniae*, and *Neisseria meningitidis* and they finally do not engulfed by phagocytosis [6, 43–46].

### 2.1. *Salmonella enterica* and Gallbladder Cancer

Gallbladder cancer is a fatal disease with notable geographical differences around the world and a tendency towards women. The main risk factor is prolonged exposure to gallstones, although bacterial infection and other inflammatory diseases are also associated [47]. Other factors include aging, low socioeconomic status, chronic infections by *S. typhi* and *H. pylori*, exposure to pollutants, heavy metals, and chemicals, and smoking in patients with identified gallbladder stones that promote cancer. These risk factors work in conjunction with an additive method which increases the incidence and accelerates the development of gallbladder cancer [48].

*Salmonella enterica* subspecies *enterica* serovar *typhi* (*Salmonella typhi*), a causative agent of typhoid fever, is colonized in the gallbladder leading to cause asymptomatic infection and gallstones, and this association is, in turn, indicated as a foremost predisposing factor for the improvement of most gallbladder cancers due to the fact that *Salmonella typhi* produces a typhoid toxin; it is probably a carcinogen, which induces the damage in DNA and alterations in cell cycle programmed in intoxicated cell [49]. Also, there is a bacterial protein known as AvrA from *Salmonella* which plays an important role in the identification of chronic infection [50]. These bacteria could survive the environment in the gallbladder by forming a biofilm which is associated with antibiotic resistance and immune system evasion and also with bacterial persistence [51]. The production of biofilms by *S. Typhi* may be a major factor in promoting persistent gallbladder infection, thus maintaining a chronic local inflammatory response and exposing the epithelium to repeated damage caused by cancer-causing toxins [49]. There are many combination factors that may induce the gallbladder cancer including cholelithiasis, genetic predisposition, slenderness, exposure to certain chemicals, reproductive factors, congenital abnormalities, and chronic infections by microorganisms; therefore, the kind of this cancer is unique [52]. Besides, the infection of *S. typhi* (typhoid) can progress leading to gallbladder cancer. Several studies demonstrated that people with *S. typhi* have increased risk of gallbladder carcinoma [53].

### 2.2. Bacterial Species and Oral Cancer

Several bacterial species in the oral cavity whether pathogenic or commensal strains have a real role in oral cancer by involving in chronic inflammation which leads to progress of oral carcinogenesis, for example, *Porphyromonas gingivalis* and *Fusobacterium nucleatum* induce the production of cytokines inflammation, proliferation of cell, inhibition of apoptosis, cellular invasion and migration, and finally alterations of cell genomic [54]. The habitat of oral cavity including 37°C temperature and pH = 6.5–7.5 of saliva represented optimum location for bacterial species; saliva is used as nutrients by the bacteria in the oral cavity [55]. Microbiome means all microorganisms in the human body; oral microbiome represents all the microorganisms in oral cavity which comprise more than 600 bacterial species [56]. Periodontal bacteria are the main pathogens of the oral cavity and the main cause of chronic periodontitis in adults, but their association with the occurrence and diagnosis of cancer is controversial [57]. Gastrointestinal carcinomas are often seen in patients suffering from periodontitis. This type of cancer may have some bacterial origin; some studies showed that the peptidyl arginine deiminase enzymes found in oral bacteria are responsible for point mutations called p53 which occurred in pancreatic cancer patients [58].

Despite the long distance between the oral cavity and the colon, they are distinguished by having a large number of distinct microbiota; studies show that bacteria in the oral cavity have the ability to be present in the colon as they change the composition of the resident bacteria leading to intestinal dysbiosis, stimulating the immune system, inflammatory response, and finally colon cancer [59]. Microbial dysbiosis is associated with many diseases, including several kinds of cancers such as colon, stomach, esophagus, pancreas, larynx, breast, and gallbladder. Cancer cells have the ability to reuse preexisting metabolic symbiosis and recycle nonmalignant cells and the resident microbiota relationships and create a new metabolic symbiosis, which leads to profound changes in the local microenvironment [60].

Despite 15% of oral cancer remaining mysterious, oral cancer is a health problem leading to high rates of mortality with several demonstrated studies about the possible role of bacteria in oral carcinoma via inhibition of apoptosis, activation of cell proliferation, promotion of cell invasion, induction of inflammation, and production cancer. Among oral bacteria, *Porphyromonas gingivalis* and *Fusobacterium nucleatum* show strong oral carcinoma in vitro and in laboratory animals [61, 62]. Currently, four common residents of the oral cavity were identified as potential bacteriostatic agents for oral carcinogenicity; these are *Porphyromonas gingivalis*, *Fusobacterium nucleatum*, *Treponema denticola*, and *Streptococcus anginosus*. They may encourage the formation of tumors and the development of oral cancer by causing chronic inflammation, promoting migration, invasion, and programmed cell inhibition, increasing cell proliferation, suppressing the immune system, and producing carcinogens [63].

### 2.3. *Helicobacter pylori* and Gastric Cancer

Gastric cancer is associated with infection by the bacteria *Helicobacter pylori* which leads to formation of lymphoma containing B cell proliferation and causing genetic abnormalities; other bacteria found to have a widespread association with carcinoma are *Salmonella typhi* in gallbladder cancer, *Chlamydia trachomotis* in cervical cancer, *Chlamydia pneumonia* and *Streptococcus bovis* in lung cancer, and *Bacteroides fragilis* and *Fusobacterium nucleatum* in colon cancer [64]. *H. pylori* is Gram-negative bacteria that colonize in stomach of 50% of people. Several studies have confirmed the relationship between these bacteria and cancer; *H. pylori* is listed as a human carcinogenic agent in 1994 by the International Agency for Research on Cancer [12, 65]. The infection with *H. pylori* causing high levels of reactive oxygen species (ROS) in gastric cancer cells compared with that in uninfected cells. Cell growth was inhibited after infection by these bacteria due to unregulated expression of pChk1 and pChk2. Infection of *H. pylori* is able to induce DNA breaks and cell cycle activation after ROS generation in gastric cancer cells [66]. The expression of cytidine deaminase, reactive of oxygen species, and reactive nitrogen species in gastric epithelial cells may be linked to *H. pylori*-related inflammation and DNA damage [67]. Aberrant DNA methylation in gastric cancer is induced by multiple driver genes and related with specific subtypes such as instability of microsatellite. Most studies showed that several types of cancer-related pathways are often altered by aberrant DNA methylation than mutations [68].

Currently, *Helicobacter pylori* is identified as the fourth common malignancy for gastric cancer and mucosa-associated lymphoid tissue (MALT). However, from all the infected people with gastritis, only 1-2% developed to gastric cancer, and the mechanisms of pathogenicity are unclear, but the possibility of microbiota in the stomach makes the link between the *Helicobacter pylori* and gastric carcinoma [69, 70]. *H. pylori* produces channel-forming toxin known as vacuolating cytotoxin A (VacA), which is unrelated to other bacterial toxins. Most of these bacteria produce this toxin by containing *vacA* gene, and it is believed that the activity of this gene is linked to the ability of bacteria to stimulate gastric cancer [71]. In addition, some studies investigated and evaluated the regulatory function of microRNAs in *H. pylori* pathogenicity especially in gastric cancer [72]. Pathogenic factors of *H. pylori*, such as cytotoxin, pathogenicity island (cag), and oncoprotein called cytotoxin-associated gene A (CagA), are involved in the carcinogenic process [73]. Furthermore, the possibility of *H. pylori* contributes to modulating the risk of developing other gastrointestinal cancers (including pancreatic, liver, esophageal, and colorectal cancers), although these associations are still not mechanically explained [74]. Therefore, the screening, treatment, and prevention of *H. pylori* colonization can decrease gastric cancer. Additional involvements that may lead to a similar effect, despite their small size, include promoting a healthy lifestyle including dietary measures, low alcohol consumption, nonsmoking, and adequate physical activity [75].

### 2.4. Gut Microbiota and Gastrointestinal Tract Cancer

Gut microbiome means all symbiotic microorganisms in the human gastrointestinal tract which defends against the pathogens and maintains the immune balance; however, any changes of the gut microbiome leading to the initiation of liver diseases include liver cancer [76]. Recently, several studies revealed that liver cancer occurred from the production of molecules by the gut microbiome (like LPS, BAs, and LTA) which contributes to the unregulated immune system in the liver [77]. There are several species of gut microbiome that may be linked and increase the risk of colon cancer by chronic infection of intestinal tissues such as *Escherichia coli* and some species of *Streptococci* [78, 79]. Colon cancer is one of the common types of cancer that arises from diet conditions and genetics. Diet changes the composition of intestinal microbiota, especially organisms that have a role in creating colon cancer such as *Bacteroides fragilis* which release toxin that induces the signal transducer which activate T-cell response, resulting in colorectal cancer [80]. Intestinal microbes have a significant effect on immune cells in the lamina propria, which affects inflammation and thus cancer. The availability of nutrients, which is the result of diet and energy balance, limits in the abundance of some energy metabolites which are important factors for epigenetic enzymes and thus affects the genetic regulation of gene expression [81]. Several studies have proposed that gut microbiota and its metabolic activities not only are linked to inducing cancer by stimulating inflammation and immune dysregulation but also interferes with the pharmacodynamics of anticancer agents [82]. Evidence confirming the ability of gut microbiota to modify the host's response to chemotherapy drugs is increasing, by three important clinical results: facilitating drug efficacy, cancellation, and waiver of anticancer effects and toxicity mediation. This implies that intestinal microorganisms are critical to the progress of personalized malignance treatment strategies [83]. Two species of bacteria *Fusobacterium nucleatum* and *E. coli* play an important role in the development and metastasis of colorectal cancer [84]. On the other hand, there are some species of bacteria that have a little role in the establishment and development of colon cancer such as *Bifidobacterium longum*, *Clostridium clostridioforme*, and *Ruminococcus* [85].

### 2.5. *Fusobacterium nucleatum* and Colorectal Cancer

Human intestinal microbiota plays a main role in human health and diseases, including colorectal cancer. Colorectal carcinogenesis is a heterogeneous process with a different set of somatic molecular changes, influenced by diet, environmental and microbial exposure, and host immunity [86]. Some bacterial species play an important role in colorectal cancer, including *Helicobacter pylori*, *Streptococcus bovis*, *Enterococcus faecalis*, *Bacteroides fragilis*, *Clostridium septicum*, *E. coli*, and *Fusobacterium* spp. [87]. *Fusobacterium* species are part of the oral gut and human intestine. Metagenomic analysis showed *Fusobacterium nucleatum* in the colon and rectal cancer tissue [86]. Many researchers have confirmed that *F. nucleatum* is clearly associated with colorectal cancer and promotes the development of colorectal tumors [88].

*Fusobacterium nucleatum* is a Gram-negative obligate anaerobic bacterium found in the oral cavity in humans, and it is involved in many diseases such as tonsillitis, sinusitis, periodontitis, gingivitis, liver abscess, and appendicitis [89–91]. *Fusobacterium nucleatum* activates the macrophages and makes it proliferate and migrate, inducing the synthesis of proinflammatory cytokines leading to colorectal cancer [92, 93]. Colorectal cancer is the fourth leading cause of cancer in worldwide, which occurs through several mechanisms including genetic, environment, life style, and role of bacterial chronic infections in development of colorectal cancer [94]. The important mechanisms of *Fusobacterium nucleatum* involved in colorectal cancer are immune modulation (such as increased myeloid-derived inhibitory cells and natural killer-inhibiting receptors), virulence factors (such as FadA and Fap2), tRNA (such as miR-21), and bacterial metabolism [95].

Failure of chemotherapy is the main cause of recurrence and poor prognosis in colorectal cancer patients. Several studies revealed that the nucleus of *Fusobacterium* was abundant in the tissues of colorectal cancer in patients with repeated chemotherapy and was associated with the patient's clinical characteristics. Moreover, our vital functional studies showed that *F. nucleatum* strengthened the colorectal cancer resistance to chemotherapy [96].

Microbiota in the intestine contributes to colorectal cancer via the pro-oncogenic activities and also via inducement of the wider bacterial community, especially its metabolome [97]. Dysbiosis means that the harmful bacteria outperform the benign bacteria, leading to diseases, including cancers [98]. Indeed, the gut microbiota undergoes many changes in composition during colorectal cancer; this indicates the main role of dysbiosis in colorectal cancer [87].

### 2.6. Microbiome and Breast Cancer

Breast cancer globally is considered to be the cause of death among women. Anatomically, the breast composed of an epithelium, stroma, and mucous immune system which form a complex microenvironment. Recently increased awareness of the role of microbes in the microenvironment has led to a series of important results for human health [99]. The commensal microorganisms associated with normal breast tissue and breast diseases are not well understood. Collectively, studies have revealed that breast tissue has a distinct microbiome with specific types fertilized in the breast tissue itself, as well as exudation of the nipple and intestinal bacteria for women with breast cancer [98].

Several studies showed the existence of diverse species of bacteria in breast tissue of healthy human [100] due to the favorable environment for the bacterial growth in breast by containing fatty tissue and extensive vasculature and lymphatic drainage [101]; these bacteria play important roles for supporting the development and immune system maturation in neonates [102]. Recent studies revealed unique microbiome in breast tissue; this microbiome varies from healthy women to breast cancer patients. The role of certain bacteria in breast cancer is complex, including the interactions between bacteria and host cells [102]. Chen et al. have shown that particular species of microbiome exist in breast tissue with bacteria in the nipple and gut in women suffering from breast cancer; these bacteria modulate the therapeutic response and are used as biomarkers for knowing the stage of breast cancer and diagnosing it [98]. It was found that women with breast cancer had higher relative abundance bacilli, *staphylococci*, and *E. coli*, isolated from breast cancer patients, and they demonstrated induction of DNA at double-stranded breaks in HeLa cells by using histon-2AX (H2AX) phosphorylation (*γ*-H2AX) assay [103]. In fact, some bacteria have been shown to help develop cancer in the lab by promoting genomic instability, invasion, and resistance to chemotherapy. However, the role of the breast microbiome in in vivo cancer appears to be more complex, as it includes many interactions between its component species and host cells [102].

## 3. Mechanisms of Carcinogenic Action

Mechanisms of bacteria which induce carcinogenesis include chronic infection as well as immune evasion and immune suppression; chronic infections alters the cell growth by disturbing the cell cycle resulting in the damage in DNA similar to that caused by genes that transformed the control of normal cells and converted them to abnormal ones [10].

Several bacterial mechanisms may influence the oncogenesis by promoting cancer through effects on transformation of cell or production of toxins; these mechanisms include deleterious alterations in the physiological host process, induction of hormones which increases the epithelial cell proliferation, and antigen-lymphoproliferation [12]. In fact, active and passive mechanisms do not depend on the strain or do not exclude each other, as bacteria may use both pathways to specifically target the tumors.

**Figure 2:**
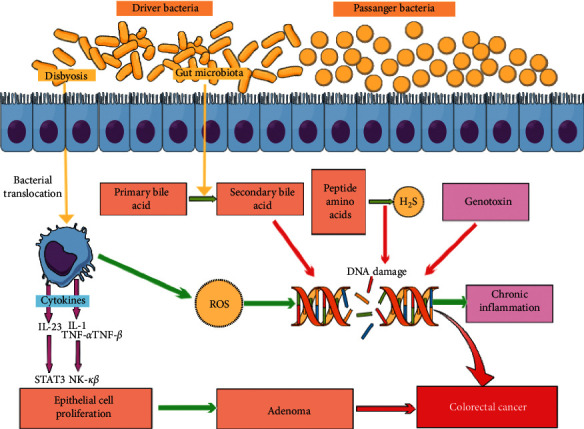
The mechanism demonstrating the role of the driver and passenger bacteria to stimulate the colorectal cancer by changing the composition of the gut microbiota (updated from [112]).

Resident microbiota and the host constitute a complex “superorganism” in which symbiotic relations give benefits to the host in several key components of life. The defects in the host's regulatory circuits that regulate bacterial sensing, or changes in the microbiome, via environmental changes, such as chronic infection, diet, or lifestyle, may disturb this symbiotic relationship and stimulate diseases [104]. The bacterial chronic infection is of great importance and can cause carcinoma in different processes; gallbladder cancer usually comes from gallstone disease in case of late diagnosis and poor treatment [105]. Chronic infections by bacteria will stimulate the immune system especially the phagocytic activity and increased oxidative stress on the contiguous cells which induce the release of oxygen radicals such as reactive oxygen (ROS) that leads to the leakage of cell membrane and DNA [1, 106]. *Salmonella typhi* is able to produce beta-glucuronidase, leading to deconjugation of conjugated toxins and bile acids; these products potentially stimulate the gallbladder carcinoma [107]. The glucuronidase enzyme was responsible for the production of intermediate substances which has the ability to bind with DNA potentially resulting in mutations [108]. On the other hand, *Chlamydia pneumoniae* invades the lung in smoking individuals according to some researches resulting in the production of nitric oxide (NO) and other oxygen radicals; all of these play an important role in lung tissue and DNA damage resulting in lung cancer [109]. Microorganisms including bacteria promote colorectal cancers by different processes such as promotion of the chronic inflammation, production of toxins, or biosynthesis of genotoxins [110]. For example, there are several carcinogenic mechanisms of *F. nucleatum*, most important of them are chronic infections, interaction of the cell surface molecules of these bacteria with the immune system, immune evasion, and immune suppression. Other mechanisms include the virulence factors of the *F. nucleatum* nucleus such as FadA, Fap2, and LPS and cell wall extracts which may act as effector molecules in the transformation of normal epithelial cells into cancerous cells [111]. The progression of colorectal cancer with driver and passenger bacteria is shown in Figure 2; the disease begins with driver bacteria to the initiation of tumorigenesis and alteration of the intestinal environment, leading to the overgrowth of passenger bacteria and finally the development of colorectal cancer [112]. The mechanism of genetic mutation which occurred from invasion by bacteria changed in the intestinal environment and damaged the DNA; carcinoma is very complex [113]. Bacteria and other microorganisms may cause infection and inflammation in tissues such as colitis, hepatitis, and gastritis which are cancers in humans at different sites because the production of nitric oxide and other oxygen radicals from infected and inflamed tissues contributes to the processing of carcinoma [109].

## 4. Bacteria in Cancer Therapy

Cancer is not an infectious disease, that is, it does not transmit from person to another, but microorganisms play an important role increasing the chance of infection. Cancer causes many physical and psychological problems for the affected patients and their families, in addition to increasing state expenses. For these reasons, estimating these novel treatments in clinical circumstances is of great importance. Treatment of cancers by conventional therapies including surgery, chemotherapy, radiotherapy, and new therapies comprising immunotherapy has increased the survival among patients. All of these therapies fight against cancer by inducing the immune system by release of inflammatory cytokines to make the immune system strong and capable of eliminating the tumors [1]. But, these therapies of cancers are difficult because there are several problems including the volume, site, stage, metastasis of tumor tissue, and nonspecific toxicity toward the normal cells. On the other hand, emerging resistance from long time exposed to conventional therapies reduces the effectiveness of chemotherapy, radiotherapy, and immunotherapy and finally losing the control of tumor [114]. The late stage of cancer that is sensitive to the conventional therapies becomes resistant later. Cancer vaccines and biological therapies are helpful to tumor cells in addition to conventional therapies, because they are characterized by less toxicity and specific targeting of tumor cells [115].

### 4.1. Bacteria Stimulate the Immune Response

Microbiota is an important factor in the progression the immune response. The interaction between the human body and resident microbiota is well balanced in healthy individuals, but its breakdown can lead to several diseases [116]. Stimulating inflammation as a result of the immune response promotes bacterial transmission to neoplastic tissue, which in turn promotes the production of inflammatory cytokines and subsequently leads to the development of tumor growth [117]. The cells of the innate immune system are located in the front of the microbiome host. These cells can sense the microorganisms or their metabolic products, translate signals into host physiological responses, and regulate microbial ecology. Distractions may contribute to the communication between the innate immune system and intestinal microbes in complex diseases [118].

For more than a century, efforts have focused primarily on amplifying the mechanisms of immune activation that humans use to eliminate invaders such as viruses and bacteria. An “immune enhancement” strategy often leads to rare objective responses and repeated immune-related adverse events (irAEs) [119].

Microbiota affects local and systemic infections. Inflammation contributes to the development and treatment of cancer, but it is still not clear whether opposing bacteria affect the inflammation in the sterile tumor microenvironment. Thus, the optimal immune response for cancer therapy requires commensal microbiota whose effects are mediated by modifying myeloid-derived cell functions in the cancer microenvironment [120]. Bacteria are regarded as antitumor agents through salvation of tumor cells by depletion of required nutrients [121] and enhancement of immune system by different mechanisms including activation of the inflammasome, for example, *Salmonella typhi* activates the pathway of inflammasome by breaking down the signals from tumor cells [122], T cell responses and release of CD4, CD25 and CD8 such as *E. coli* are able to degrade of tumor cells by induce to production of T cell and release of CD8 [123], release of TNF-*α* induced by *Salmonella enterica* could degrade the tumor cells via the initiation of blood flow into tumors [124].*Salmonella enterica* is a facultative anaerobic bacterium characterized by its ability to colonized and proliferate inside the macrophage and dendritic cells, therefore used for vaccination [125]. Previous studies showed that antigens of *Helicobacter pylori* activate the NK cells to secrete IFN-*γ*. There is also a noticeable synergistic effect in NK cells stimulated by bacterial lysate and low levels of IL-12, which is the cytokine produced by macrophages and dendritic cells in the stomach infected with *H. pylori,* which causes at least half of the gastric cancers [126].

### 4.2. Oncolytic Bacteria

For more than a century, a group of researchers have revealed the possibility of using bacteria to kill cancerous tumors. This treatment causes an immune response that rejects the tumor and protects the patient from recurrence of the disease. Then, another group of researchers used different bacteria to test their antitumor activity in animal models and patients. The basis for these tests indicates an innate immune response that is activated by bacteria. Finally, various publications covered many aspects of oncolytic bacteria [127].

**Figure 3:**
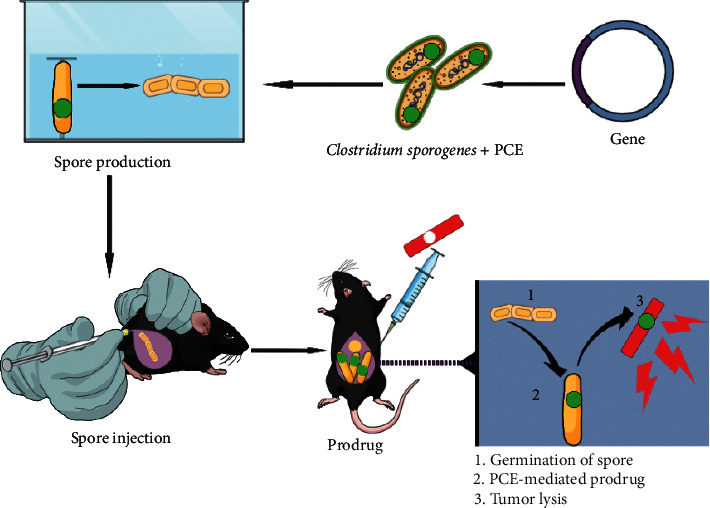
Engineered *Clostridium sporogenes* playing important roles as a delivery vector. In the beginning, the gene nitroreductase (PCE) is introduced into the chromosome of bacteria. Second, the engineered strains are put in specific environment to form spores. Third, the spores are injected into the lab mice infected with cancer. Forth, the spores colonize and the prodrug is transferred into the tumor cells causing tumor lysis (updated from [129]).

Bacteria have many benefits compared with the traditional treatments, for example, *Clostridium* spp. can grow and proliferate in the tumor cells because it provides anaerobic conditions and adequate metabolic nutrients [17]. *Clostridium novyi* is a wild strain that has the ability to remove the lethal toxin gene through inhibiting the phage by carrying the gene existing in the spores; then, the spores will grow perfectly into the tumor, resulting in the destruction of the tumor [17, 128]. *Clostridium* spp. is obligate anaerobic bacteria that colonize and proliferate in necrotic regions of solid tumor because these bacteria forming endospores are of capable germinating in these regions; therefore, *Clostridium* species are regarded as tumor delivery agents for cancer therapy (Figure 3) [129].

Rolim et al. observed that the bacterial activities reduce the development of tumors by several methods such as regulation the effects on environment of the tumor, change the tumor receptors, starvation and suffocation of cancer cells, enzymes and toxins secreted by bacteria, and genetic modification [130] Nonpathogenic bacteria after genetically modification are favorites for potential antitumor agents by direct effects or excreted molecules [131]. When *Salmonella* was administrated into the solid tumor within hemorrhagic area, the bacteria will proliferate and consume the oxygen and nutrients, leading to necrotic regions of hemorrhaging area, destroy the blood vessels, and finally reduce the proliferation of tumor cells [34, 124]. Most bacteria have the ability to attack and colonize cancer cells, and in best cases, this leads to the treatment of cancer completely [132–134]. The recent studies demonstrated that the microbiome within the human body targeted the tumor cells because of decreased immune activity in the necrotic cores of tumor [135]; some bacteria can grow into tumors that are preferred for reproduction by consuming food and oxygen, causing cancer cells to starve and suffocate. This phenomenon opened the doors to the possibility of using nonpathogenic bacteria to deliver drugs into the tumor [136].

**Figure 4:**
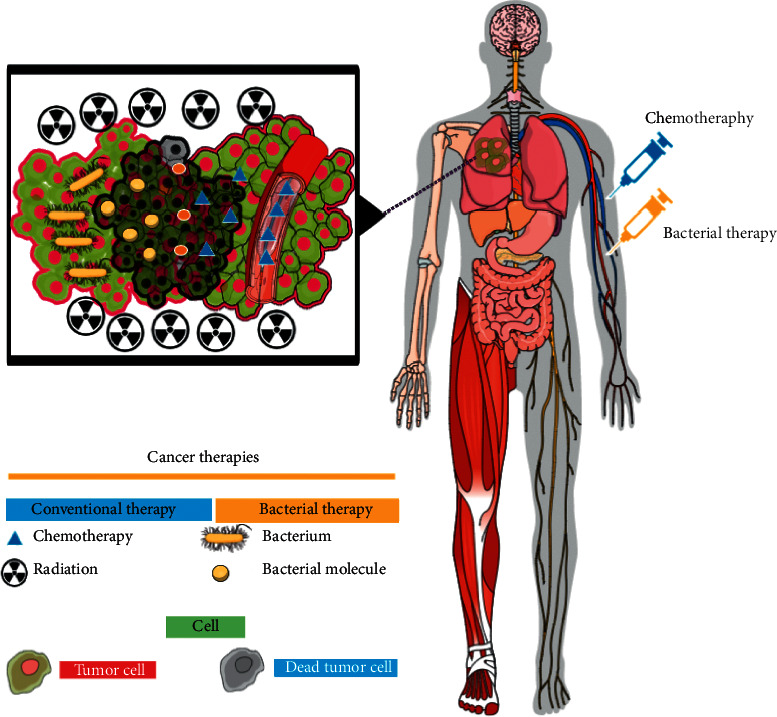
The difference between the types of cancerous treatments. Chemotherapy has a limited ability to penetrate neoplastic cells because it works by passive transport during the cell membrane compared with bacteria that can overcome these barriers by colonizing, increasing the proliferation and immune stimulation in cancer environment (updated from [7]).

It is a true fact that cancer therapy and bacterial treatment are taken independently (Figure 4), so Singh et al. and his coworkers invented a dual drug called dualsome that eliminates cancer and at the same time gets rid of the curative bacteria in the cancerous tissue. Dualsome consists of three parts folic acid attached to the surface for imparting the cancer cell, antibacterial peptides on the surface such as sushiS3, and in the core, liposomes loaded with cancer conventional therapy (doxorubicin) [137].

### 4.3. Engineered Bacteria to Fight Cancer

Programmed bacteria become the appropriate and unique solution to these challenges [21]. Therefore, the other treatments of cancers are very necessary; therapeutic bacteria are one of these treatments able to defeat some of the problems of conventional cancer therapies. Bacteria play important roles as antitumorigenic agents by whole bacteria or cytotoxin or peptides carried by them [20]. One of effective cancer therapies is the use of toxins and spores of bacteria to eliminate the tumor cells. Since bacterial spores grow well in necrotic tissue and in anaerobic conditions, bacterial spores have been used to treat cancer. They play an important role in killing tumor cells or converting cancerous cells and returning them to normal [138].

Genetic engineering is a technique to insert modification material into the organism or alter the genetic material of the original organism. Genetic engineering of microorganisms is a new way to treat cancer because the studies that pertain to microbiome indicate an increase in the number of bacteria in the tumor tissue [135]. In fact, the presence of blood vessels in the cancerous tissue despite its irregular organization, it provides chance of survival from disease and the growth and proliferation of attenuated bacteria through the availability of nutrients [139, 140].

In 1868, the physician W. Busch observed regression of tumors in patients when they had a skin infection (erysipelas); he performed an experiment in which he chose a woman with cancer and put her on a tainted bed with *Streptococcus pyogenes* where he noticed that she had recovered from cancer, but that she remained infected with bacteria [141].

In 1891, the physician Dr. William Coley was the first to use the bacteria and their toxins in the treatment of cases ending with cancer; he used live *Streptococcus pyogenes* and killed them by heating and injected them into his patients. After that, Dr. Coley was able to develop a safe vaccine composed of two of bacterial species after killing them that include *Streptococcus pyogenes* and *Serratia marcescens*; the vaccine was later called Coley's vaccine “Coley's toxin” which was widely used for different types of cancers such as carcinomas and lymphomas [10, 142, 143]. In initial tests, the bacterium *Salmonella* was injected into the solid tumor, where it was observed that the cancer cells were killed. However, bacterial injection is considered unsafe for patients with immunocompromised tumor [144].

In 1976, bacterial cancer therapy by using Bacillus Calmette-Guerin (BCG) was established. The researchers Morales, Eidinger, and Bruce in this year used the attenuated *Mycobacterium bovis* for the treatment of bladder cancer successfully [145].

In the past, living bacteria including *Streptococci* and *Clostridia* were first used by clinicians for cancer therapy and to promote the survival in the animal models, after which genetic engineering was introduced for modifying the bacteria to convert it for bacterial therapies by different mechanisms including native toxin of bacteria, combination with other treatment, anticancer agents, gene transfer, expression of antigens, interference of RNA, and cleavage of prodrug [7, 146]. There are many genetically modified bacterial genera of the most important species, *Salmonella*, *Clostridium*, *Lactobacilli*, *E. coli*, *Bifidobacterium*, *Pseudomonas*, *Streptococcus*, *Proteus*, *Caulobacter*, and *Listeria* [20]. For example, *Salmonella typhi* and the materials that are derived from it can be used directly as antitumor agent and as vaccine [147].

Despite the successful use of weakened bacterial strains in cancer treatment, it does have many side effects, so it has been used probiotic such as *E. coli* Nissle 1971; it is very safe and without virulence genes [148]. Another probiotic is lactobacilli [149].

Recently, the world has turned to a new hope for the treatment of cancer by using engineering bacteria to carry special antibodies that have the ability to distinguish cancer cells. Among the cancer cells, there is a very dangerous subpopulation group with the ability to renew and differentiate into any of cell types causing tumor relapse [150]. Therefore, it is necessary to develop therapies targeting these subpopulations, the most important of which is genetically engineered bacteria including Trojan-horse bacteria that have the ability to express markers on tumor cells and dormant cells [151, 152].

It is necessary to genetically modify the bacteria before using it as a cancer therapy to reduce its pathological effect and increase its effectiveness to remove tumor cells. Bacterial therapies remove malignant cells in several ways, including the production of substances or stimulation of the immune response to cause inflammation [153].

There are several and unique mechanisms by which the bacteria target tumor tissue, the best example is using attenuated light-emitting *Salmonella typhimurium* wild strains that were defective in ppGpp synthesis, a group of researchers clearly established that bacteria accumulate entirely in tumors after intravenously injected in different types of tumor-bearing mice [154–157].

## 5. Conclusion

We have demonstrated the prominent ways in which bacteria can modify the formation of tumors. Besides stimulating cancer by disrupting the host's normal defense processes such as inflammation and antigen recognition, some bacteria have also shown the production of tumor proteins by products of metabolism that have direct myogenic or mutagenic effects. There are also other potential mechanisms that we have mentioned, such as the role of bacterial infections as cofactors in the development of metastasis. Although conventional cancer treatments are still the dominant treatments, bacterial therapy has shown noticeable effects, due to its high specificity, its ability to control after ingestion, and its condition in many live studies. The method of bacteria targeting tumors is an ideal way to deliver therapeutic loads because of the tumor selectivity and its wide gene packaging capacity. However, there are still many problems for the use of bacteria in clinical practice as antitumor agents including bacterial toxicity, DNA instability, limited targeting efficiency, selection of safe and practical bacterial strains, and combination testing with other treatments. In spite of the great curative potential of engineered tumor-targeting bacteria, a successful treatment of cancer still expects a combination approach soon, because the heterogeneity of cancer, at the molecular and histological levels, makes it very difficult to achieve treatment using single anticancer agents. Although additional studies are needed to explain why bacteria are useful in targeting and growing tumors, it cannot be denied that the therapeutic ability of bacteria to target, penetrate, and reproduce in tumors is a promising feature that overcomes some of the current limitations of conventional treatments. Focusing on bacterial therapy by using genetically engineered bacteria alone or with conventional cancer treatment opens the doors for researchers to develop this treatment free of side effects, and it is possible to save humanity from cancer permanently.

In summary, it is expected that, in addition to the intrinsic antitumor effects, bacterial infection makes its most important involvement in tumor regression by activating a complex set of immune cells. Although the basic mechanism varies, bacteria are likely to offer a unique immunotherapy treatment strategy that can be enhanced by the advanced genetic engineering of bacterial strains.

## Data Availability

Data used to support the findings of this study are included within the review article.

## References

[1] Wheeler Torres V. L., Olivar L. C., Navarro C. (2018). Bacteria in cancer therapy: beyond immunostimulation. *Journal of Cancer Metastasis and Treatment*.

[2] Hoadley K. A., Yau C., Wolf D. M. (2014). Multiplatform analysis of 12 cancer types reveals molecular classification within and across tissues of origin. *Cell*.

[3] Ashu E. E., Xu J., Yuan Z.-C. (2019). Bacteria in cancer therapeutics: a framework for effective therapeutic bacterial screening and identification. *Journal of Cancer*.

[4] Blot W. J., McLaughlin J. K., Winn D. M. (1988). Smoking and drinking in relation to oral and pharyngeal cancer. *Cancer Research*.

[5] Parkin D. M., Mesher D., Sasieni P. (2011). 10. Cancers attributable to dietary factors in the UK in 2010. *British Journal of Cancer*.

[6] van Elsland D., Neefjes J. (2018). Bacterial infections and cancer. *EMBO Reports*.

[7] Sedighi M., Bialvaei A. Z., Hamblin M. R. (2019). Therapeutic bacteria to combat cancer; current advances, challenges, and opportunities. *Cancer Medicine*.

[8] Karpiński T. M. (2019). Role of oral microbiota in cancer development. *Microorganisms*.

[9] Dong Q.-l., Xing X.-y. (2018). Cancer cells arise from bacteria. *Cancer Cell International*.

[10] Mager D. L. (2006). Bacteria and cancer: cause, coincidence or cure? A review. *Journal of Translational Medicine*.

[11] Vedham V., Divi R. L., Starks V. L., Verma M. (2014). Multiple infections and cancer: implications in epidemiology. *Technology in Cancer Research & Treatment*.

[12] Chang A. H., Parsonnet J. (2010). Role of bacteria in oncogenesis. *Clinical Microbiology Reviews*.

[13] Coussens L. M., Werb Z. (2002). Inflammation and cancer. *Nature*.

[14] Sethi V., Vitiello G. A., Saxena D., Miller G., Dudeja V. (2019). The role of the microbiome in immunologic development and its implication for pancreatic cancer immunotherapy. *Gastroenterology*.

[15] Reis S. A. d., da Conceição L. L., Peluzio M. d. C. G. (2019). Intestinal microbiota and colorectal cancer: changes in the intestinal microenvironment and their relation to the disease. *Journal of Medical Microbiology*.

[16] Spratt D. E., Pei X., Yamada J., Kollmeier M. A., Cox B., Zelefsky M. J. (2013). Long-term survival and toxicity in patients treated with high-dose intensity modulated radiation therapy for localized prostate cancer. *International Journal of Radiation Oncology∗Biology∗Physics*.

[17] Nair N., Kasai T., Seno M. (2014). Bacteria: prospective savior in battle against cancer. *Anticancer Research*.

[18] Nguyen V. H., Min J.-J. (2017). Salmonella-mediated cancer therapy: roles and potential. *Nuclear Medicine and Molecular Imaging*.

[19] Pangilinan C. R., Lee C. H. (2019). Salmonella-based targeted cancer therapy: updates on a promising and innovative tumor immunotherapeutic strategy. *Biomedicines*.

[20] Nallar S. C., Xu D.-Q., Kalvakolanu D. V. (2017). Bacteria and genetically modified bacteria as cancer therapeutics: current advances and challenges. *Cytokine*.

[21] Zhou S., Gravekamp C., Bermudes D., Liu K. (2018). Tumour-targeting bacteria engineered to fight cancer. *Nature Reviews Cancer*.

[22] Maslowski K. M., Takahashi M., Nakanishi Y. (2019). Attenuated Salmonella typhimurium cancer therapy has direct effects on the tumor epithelium in colorectal cancer. *Biorxiv*.

[23] Nauts H. C. (1980). The beneficial effects of bacterial infections on host resistance to cancer. End results in 449 cases. A study and abstracts of reports in the world medical literature (1775–1980) and personal communication. *Cancer Research Institute Monograph*.

[24] Dzutsev A., Badger J. H., Perez-Chanona E. (2017). Microbes and cancer. *Annual Review of Immunology*.

[25] Laliani G., Ghasemian Sorboni S., Lari R. (2020). Bacteria and cancer: different sides of the same coin. *Life Sciences*.

[26] Firor W. M., Gey G. O. (1945). Observations on the conversion of normal into malignant cells. *Annals of Surgery*.

[27] Borek C. (1980). X-ray-induced in vitro neoplastic transformation of human diploid cells. *Nature*.

[28] Milo G. E., DiPaolo J. A. (1978). Neoplastic transformation of human diploid cells in vitro after chemical carcinogen treatment. *Nature*.

[29] Sachs L., Medina D. (1961). In vitro transformation of normal cells by polyoma virus. *Nature*.

[30] Morales-Sánchez A., Fuentes-Pananá E. (2014). Human viruses and cancer. *Viruses*.

[31] Lax A. J. (2005). Opinion: bacterial toxins and cancer—case to answer?. *Nature Reviews Microbiology*.

[32] Schoppmann S. F., Birner P., Stöckl J. (2002). Tumor-associated macrophages express lymphatic endothelial growth factors and are related to peritumoral lymphangiogenesis. *The American Journal of Pathology*.

[33] Baik S. C., Youn H. S., Chung M. H. (1996). Increased oxidative DNA damage in Helicobacter pylori-infected human gastric mucosa. *Cancer Research*.

[34] Song S., Vuai M. S., Zhong M. (2018). The role of bacteria in cancer therapy—enemies in the past, but allies at present. *Infectious Agents and Cancer*.

[35] de Martel C., Ferlay J., Franceschi S. (2012). Global burden of cancers attributable to infections in 2008: a review and synthetic analysis. *The Lancet Oncology*.

[36] Mc C. W., Mason J. M. (1951). Enterococcal endocarditis associated with carcinoma of the sigmoid; report of a case. *Journal of the Medical Association of the State of Alabama*.

[37] Nath G., Gulati A. K., Shukla V. K. (2010). Role of bacteria in carcinogenesis, with special reference to carcinoma of the gallbladder. *World Journal of Gastroenterology*.

[38] Russell W. (1890). An address on a characteristic organism of cancer. *BMJ*.

[39] Glover T. (1926). Progress in cancer research. *Canada Lancet and Practitioner*.

[40] L’esperance E. S. (1931). Studies IN Hodgkin’s disease. *Annals of Surgery*.

[41] Mazet G. (1941). *Etude Bacteriologique sur la Maladie d’Hodgkin*.

[42] Diller I. C. (1974). Tumor incidence in ICR/albino and C57/B16JNIcr male mice injected with organisms cultured from mouse malignant tissue. *Growth*.

[43] Pluschke G., Mayden J., Achtman M., Levine R. P. (1983). Role of the capsule and the O antigen in resistance of O18: K1 *Escherichia coli* to complement-mediated killing. *Infection and Immunity*.

[44] Abeyta M., Hardy G. G., Yother J. (2003). Genetic alteration of capsule type but not PspA type affects accessibility of surface-bound complement and surface antigens of Streptococcus pneumoniae. *Infection and Immunity*.

[45] Brown E. J., Hosea S. W., Frank M. M. (1983). The role of antibody and complement in the reticuloendothelial clearance of pneumococci from the bloodstream. *Clinical Infectious Diseases*.

[46] Winkelstein J. A., Tomasz A. (1978). Activation of the alternative complement pathway by pneumococcal cell wall teichoic acid. *Journal of Immunology*.

[47] Espinoza J. A., Bizama C., García P. (2016). The inflammatory inception of gallbladder cancer. *Biochimica et Biophysica Acta (BBA)—Reviews on Cancer*.

[48] Dutta U., Bush N., Kalsi D., Popli P., Kapoor V. K. (2019). Epidemiology of gallbladder cancer in India. *Chinese Clinical Oncology*.

[49] Di Domenico E. G., Cavallo I., Pontone M., Toma L., Ensoli F. (2017). Biofilm producing Salmonella typhi: chronic colonization and development of gallbladder cancer. *International Journal of Molecular Sciences*.

[50] Lu R., Bosland M., Xia Y., Zhang Y.-g., Kato I., Sun J. (2017). Presence of Salmonella AvrA in colorectal tumor and its precursor lesions in mouse intestine and human specimens. *Oncotarget*.

[51] Chin K. C. J., Taylor T. D., Hebrard M., Anbalagan K., Dashti M. G., Phua K. K. (2017). Transcriptomic study of *Salmonella enterica* subspecies enterica serovar Typhi biofilm. *BMC Genomics*.

[52] Wistuba I. I., Gazdar A. F. (2004). Gallbladder cancer: lessons from a rare tumour. *Nature Reviews Cancer*.

[53] Welton J., Marr J., Friedman S. (1979). Association between hepatobiliary cancer and typhoid carrier state. *The Lancet*.

[54] Chattopadhyay I., Verma M., Panda M. (2019). Role of oral microbiome signatures in diagnosis and prognosis of oral cancer. *Technology in Cancer Research & Treatment*.

[55] Takahashi N. (2005). Microbial ecosystem in the oral cavity: metabolic diversity in an ecological niche and its relationship with oral diseases. *International Congress Series*.

[56] Dewhirst F. E., Chen T., Izard J. (2010). The human oral microbiome. *Journal of Bacteriology*.

[57] Xiao L., Zhang Q., Peng Y., Wang D., Liu Y. (2020). The effect of periodontal bacteria infection on incidence and prognosis of cancer: a systematic review and meta-analysis. *Medicine*.

[58] Öğt avai M. (2015). Oral bacteria in pancreatic cancer: mutagenesis of the p53 tumour suppressor gene. *International Journal of Clinical and Experimental Pathology*.

[59] Koliarakis I., Messaritakis I., Nikolouzakis T. K., Hamilos G., Souglakos J., Tsiaoussis J. (2019). Oral bacteria and intestinal dysbiosis in colorectal cancer. *International Journal of Molecular Sciences*.

[60] Nunes S. C., Serpa J. (2020). Recycling the interspecific relations with epithelial cells: bacteria and cancer metabolic symbiosis. *Advances in Experimental Medicine and Biology*.

[61] Perera M., Al-hebshi N. N., Speicher D. J., Perera I., Johnson N. W. (2016). Emerging role of bacteria in oral carcinogenesis: a review with special reference to perio-pathogenic bacteria. *Journal of Oral Microbiology*.

[62] Rubinstein M. R., Wang X., Liu W., Hao Y., Cai G., Han Y. W. (2013). *Fusobacterium nucleatum* promotes colorectal carcinogenesis by modulating E-cadherin/beta-catenin signaling via its FadA adhesin. *Cell Host & Microbe*.

[63] Zhang W.-l., Wang S.-s., Wang H.-f., Tang Y.-J., Tang Y.-l., Liang X.-h. (2019). Who is who in oral cancer?. *Experimental Cell Research*.

[64] Rajeev R., Choudhary K., Panda S., Gandhi N. (2012). Role of bacteria in oral carcinogenesis. *South Asian Journal of Cancer*.

[65] (1994). International Agency for Research on Cancer. *IARC Working Group on the Evaluation of Carcinogenic Risks to Humans: Schistosomes*.

[66] Shi Y., Wang P., Guo Y., Liang X., Li Y., Ding S. (2019). Helicobacter pylori-induced DNA damage is a potential driver for human gastric cancer AGS cells. *DNA and Cell Biology*.

[67] Shimizu T., Chiba T., Marusawa H. (2017). Helicobacter pylori-mediated genetic instability and gastric carcinogenesis. *Current Topics in Microbiology and Immunology*.

[68] Maeda M., Moro H., Ushijima T. (2017). Mechanisms for the induction of gastric cancer by *Helicobacter pylori* infection: aberrant DNA methylation pathway. *Gastric Cancer*.

[69] Dias-Jacome E., Libânio D., Borges-Canha M., Galaghar A., Pimentel-Nunes P. (2016). Gastric microbiota and carcinogenesis: the role of non-Helicobacter pylori bacteria—a systematic review. *Revista Española de Enfermedades Digestivas*.

[70] Herrera V., Parsonnet J. (2009). *Helicobacter pylori* and gastric adenocarcinoma. *Clinical Microbiology and Infection*.

[71] McClain M. S., Beckett A. C., Cover T. L. (2017). *Helicobacter pylori* vacuolating toxin and gastric cancer. *Toxins*.

[72] Dastmalchi N., Safaralizadeh R., Banan Khojasteh S. M. (2019). The correlation between microRNAs and *Helicobacter pylori* in gastric cancer. *Pathogens and Disease*.

[73] Mégraud F., Bessède E., Varon C. (2015). *Helicobacter pylori* infection and gastric carcinoma. *Clinical Microbiology and Infection*.

[74] Venerito M., Vasapolli R., Malfertheiner P. (2016). *Helicobacter pylori* and gastric cancer: timing and impact of preventive measures. *Advances in Experimental Medicine and Biology*.

[75] den Hoed C. M., Kuipers E. J. (2016). Gastric cancer: how can we reduce the incidence of this disease?. *Current Gastroenterology Reports*.

[76] Jia B., Jeon C. O. (2019). Promotion and induction of liver cancer by gut microbiome-mediated modulation of bile acids. *PLOS Pathogens*.

[77] Ma C., Han M., Heinrich B. (2018). Gut microbiome-mediated bile acid metabolism regulates liver cancer via NKT cells. *Science*.

[78] Kim N. H., Park J. P., Jeon S. H. (2002). Purulent pericarditis caused by group G streptococcus as an initial presentation of colon cancer. *Journal of Korean Medical Science*.

[79] Siegert C. E., Overbosch D. (1995). Carcinoma of the colon presenting as Streptococcus sanguis bacteremia. *The American Journal of Gastroenterology*.

[80] Wu S., Rhee K.-J., Albesiano E. (2009). A human colonic commensal promotes colon tumorigenesis via activation of T helper type 17 T cell responses. *Nature Medicine*.

[81] Bultman S. J. (2017). Interplay between diet, gut microbiota, epigenetic events, and colorectal cancer. *Molecular Nutrition & Food Research*.

[82] Meng C., Bai C., Brown T. D., Hood L. E., Tian Q. (2018). Human gut microbiota and gastrointestinal cancer. *Genomics, Proteomics & Bioinformatics*.

[83] Alexander J. L., Wilson I. D., Teare J., Marchesi J. R., Nicholson J. K., Kinross J. M. (2017). Gut microbiota modulation of chemotherapy efficacy and toxicity. *Nature Reviews Gastroenterology & Hepatology*.

[84] Castellarin M., Warren R. L., Freeman J. D. (2012). *Fusobacterium nucleatum* infection is prevalent in human colorectal carcinoma. *Genome Research*.

[85] Sobhani I., Amiot A., Le Baleur Y. (2013). Microbial dysbiosis and colon carcinogenesis: could colon cancer be considered a bacteria-related disease?. *Therapeutic Advances in Gastroenterology*.

[86] Nosho K. (2016). Association of *Fusobacterium nucleatum* with immunity and molecular alterations in colorectal cancer. *World Journal of Gastroenterology*.

[87] Gagnière J. (2016). Gut microbiota imbalance and colorectal cancer. *World Journal of Gastroenterology*.

[88] Shang F.-M., Liu H.-L. (2018). *Fusobacterium nucleatum* and colorectal cancer: a review. *World Journal of Gastrointestinal Oncology*.

[89] Yang Y., Misra B. B., Liang L. (2019). Integrated microbiome and metabolome analysis reveals a novel interplay between commensal bacteria and metabolites in colorectal cancer. *Theranostics*.

[90] Yu J., Feng Q., Wong S. H. (2017). Metagenomic analysis of faecal microbiome as a tool towards targeted non-invasive biomarkers for colorectal cancer. *Gut*.

[91] Ganesan K., Guo S., Fayyaz S., Zhang G., Xu B. (2019). Targeting programmed *Fusobacterium nucleatum* Fap2 for colorectal cancer therapy. *Cancers*.

[92] Strauss J., Kaplan G. G., Beck P. L. (2011). Invasive potential of gut mucosa-derived *Fusobacterium nucleatum* positively correlates with IBD status of the host. *Inflammatory Bowel Diseases*.

[93] Manson McGuire A., Cochrane K., Griggs A. D. (2014). Evolution of invasion in a diverse set of *Fusobacterium* species. *mBio*.

[94] Lucas C., Barnich N., Nguyen H. T. T. (2017). Microbiota, inflammation and colorectal cancer. *International Journal of Molecular Sciences*.

[95] Hashemi Goradel N., Heidarzadeh S., Jahangiri S. (2019). *Fusobacterium nucleatum* and colorectal cancer: a mechanistic overview. *Journal of Cellular Physiology*.

[96] Yu T., Guo F., Yu Y. (2017). *Fusobacterium nucleatum* promotes chemoresistance to colorectal cancer by modulating autophagy. *Cell*.

[97] Louis P., Hold G. L., Flint H. J. (2014). The gut microbiota, bacterial metabolites and colorectal cancer. *Nature Reviews Microbiology*.

[98] Chen J., Douglass J., Prasath V. (2019). The microbiome and breast cancer: a review. *Breast Cancer Research and Treatment*.

[99] Hieken T. J., Chen J., Hoskin T. L. (2016). The microbiome of aseptically collected human breast tissue in benign and malignant disease. *Scientific Reports*.

[100] Thompson K. J., Ingle J. N., Tang X. (2017). A comprehensive analysis of breast cancer microbiota and host gene expression. *PLoS One*.

[101] Urbaniak C., Cummins J., Brackstone M. (2014). Microbiota of human breast tissue. *Applied and Environmental Microbiology*.

[102] O’Connor H., MacSharry J., Bueso Y. F. (2018). Resident bacteria in breast cancer tissue: pathogenic agents or harmless commensals?. *Discovery Medicine*.

[103] Urbaniak C., Gloor G. B., Brackstone M., Scott L., Tangney M., Reid G. (2016). The microbiota of breast tissue and its association with breast cancer. *Applied and Environmental Microbiology*.

[104] Schwabe R. F., Jobin C. (2013). The microbiome and cancer. *Nature Reviews Cancer*.

[105] Lazcano-Ponce E. C., Miquel J. F., Munoz N. (2001). Epidemiology and molecular pathology of gallbladder cancer. *CA: A Cancer Journal for Clinicians*.

[106] Krishnan S., Eslick G. D. (2014). Streptococcus bovis infection and colorectal neoplasia: a meta-analysis. *Colorectal Disease*.

[107] Hill M. J. (1995). Chronic bacterial infection and subsequent human carcinogenesis. *European Journal of Cancer Prevention: The Official Journal of the European Cancer Prevention Organisation (ECP)*.

[108] Kinoshita N., Gelboin H. (1978). Beta-Glucuronidase catalyzed hydrolysis of benzo(a)pyrene-3-glucuronide and binding to DNA. *Science*.

[109] Ohshima H., Bartsch H. (1994). Chronic infections and inflammatory processes as cancer risk factors: possible role of nitric oxide in carcinogenesis. *Mutation Research/Fundamental and Molecular Mechanisms of Mutagenesis*.

[110] Candela M., Turroni S., Biagi E. (2014). Inflammation and colorectal cancer, when microbiota-host mutualism breaks. *World Journal of Gastroenterology*.

[111] Gholizadeh P., Eslami H., Kafil H. S. (2017). Carcinogenesis mechanisms of *Fusobacterium nucleatum*. *Biomedicine & Pharmacotherapy*.

[112] Nistal E., Fernández-Fernández N., Vivas S., Olcoz J. L. (2015). Factors determining colorectal cancer: the role of the intestinal microbiota. *Frontiers in Oncology*.

[113] Ou J., Carbonero F., Zoetendal E. G. (2013). Diet, microbiota, and microbial metabolites in colon cancer risk in rural Africans and African Americans. *The American Journal of Clinical Nutrition*.

[114] Datta N. R., Ordóñez S. G., Gaipl U. S. (2015). Local hyperthermia combined with radiotherapy and-/or chemotherapy: recent advances and promises for the future. *Cancer Treatment Reviews*.

[115] Sarotra P., Medhi B. (2016). Use of bacteria in cancer therapy. *Recent Results in Cancer Research*.

[116] Kamada N., Núñez G. (2014). Regulation of the immune system by the resident intestinal bacteria. *Gastroenterology*.

[117] Shiotani A., Fukushima S., Matsumoto H. (2019). Carcinogenesis and gut microbiota. *Gan To Kagaku Ryoho*.

[118] Thaiss C. A., Zmora N., Levy M., Elinav E. (2016). The microbiome and innate immunity. *Nature*.

[119] Sanmamed M. F., Chen L. (2018). A paradigm shift in cancer immunotherapy: from enhancement to normalization. *Cell*.

[120] Iida N., Dzutsev A., Stewart C. A. (2013). Commensal bacteria control cancer response to therapy by modulating the tumor microenvironment. *Science*.

[121] Danino T., Prindle A., Hasty J., Bhatia S. (2013). Measuring growth and gene expression dynamics of tumor-targeted *S. typhimurium* bacteria. *Journal of Visualized Experiments: JoVE*.

[122] Phan T. X., Nguyen V. H., Duong M. T.-Q., Hong Y., Choy H. E., Min J.-J. (2015). Activation of inflammasome by attenuated *Salmonella typhimurium* in bacteria-mediated cancer therapy. *Microbiology and Immunology*.

[123] Stern C., Kasnitz N., Kocijancic D. (2015). Induction of CD4^+^ and CD8^+^ anti-tumor effector T cell responses by bacteria mediated tumor therapy. *International Journal of Cancer*.

[124] Leschner S., Westphal K., Dietrich N. (2009). Tumor invasion of *Salmonella enterica* serovar Typhimurium is accompanied by strong hemorrhage promoted by TNF-alpha. *PLoS One*.

[125] Hernandez-Luna M. A., Luria-Perez R., Huerta-Yepez S. (2013). Therapeutic intervention alternatives in cancer, using attenuated live bacterial vectors: *Salmonella enterica* as a carrier of heterologous molecules. *Revista de Investigación Clínica*.

[126] Lindgren Å., Yun C.-H., Sjöling Å. (2011). Impaired IFN-*γ* production after stimulation with bacterial components by natural killer cells from gastric cancer patients. *Experimental Cell Research*.

[127] Rius-Rocabert S., Pinel F. L., Pozuelo M. J., García A., Nistal-Villan E. (2019). Oncolytic bacteria: past, present and future. *FEMS Microbiology Letters*.

[128] Dang L. H., Bettegowda C., Huso D. L., Kinzler K. W., Vogelstein B. (2001). Combination bacteriolytic therapy for the treatment of experimental tumors. *Proceedings of the National Academy of Sciences*.

[129] Kubiak A. M., Minton N. P. (2015). The potential of clostridial spores as therapeutic delivery vehicles in tumour therapy. *Research in Microbiology*.

[130] Rolim M. S. L., Montenegro Y. H. A., Carneiro A. M., Ceballos B. S. O. (2019). Bacteria and cancer: advances, perspectives and applications. *Clinical Microbiology and Infectious Diseases*.

[131] Patyar S., Joshi R., Byrav D. P., Prakash A., Medhi B., Das B. (2010). Bacteria in cancer therapy: a novel experimental strategy. *Journal of Biomedical Science*.

[132] Forbes N. S. (2006). Profile of a bacterial tumor killer. *Nature Biotechnology*.

[133] Roberts N. J., Zhang L., Janku F. (2014). Intratumoral injection of Clostridium novyi-NT spores induces antitumor responses. *Science Translational Medicine*.

[134] Hatzikirou H., López Alfonso J. C., Leschner S., Weiss S., Meyer-Hermann M. (2017). Therapeutic potential of bacteria against solid tumors. *Cancer Research*.

[135] Harimoto T., Danino T. (2019). Engineering bacteria for cancer therapy. *Emerging Topics in Life Sciences*.

[136] Alderton G. (2019). Synthetic bacterial cancer therapy. *Science*.

[137] Singh R., Kumar C. S., Banerjee M., Gupta S. (2019). A dual drug delivery platform for cancer-bacteria cotargeting. *ACS Applied Bio Materials*.

[138] Weerakkody L. R., Witharana C. (2019). The role of bacterial toxins and spores in cancer therapy. *Life Sciences*.

[139] Forbes N. S., Munn L. L., Fukumura D., Jain R. K. (2003). Sparse initial entrapment of systemically injected *Salmonella typhimurium* leads to heterogeneous accumulation within tumors. *Cancer Research*.

[140] Kramer M. G., Masner M., Ferreira F. A., Hoffman R. M. (2018). Bacterial therapy of cancer: promises, limitations, and insights for future directions. *Frontiers in Microbiology*.

[141] Oelschlaeger T. A. (2010). Bacteria as tumor therapeutics?. *Bioengineered Bugs*.

[142] Richardson M. A., Ramirez T., Russell N. C., Moye L. A. (1999). Coley toxins immunotherapy: a retrospective review. *Alternative Therapies in Health and Medicine*.

[143] Nybo K. (2018). Part I: fighting cancer with deadly bacteria. *BioTechniques*.

[144] Clairmont C., Lee K. C., Pike J. (2000). Biodistribution and genetic stability of the novel antitumor agent VNP20009, a genetically modified strain of *Salmonella typhimuvium*. *The Journal of Infectious Diseases*.

[145] Morales A., Eidinger D., Bruce A. W. (2002). Intracavitary Bacillus Calmette-Guerin in the treatment of superficial bladder tumors. *Journal of Urology*.

[146] Forbes N. S. (2010). Engineering the perfect (bacterial) cancer therapy. *Nature Reviews Cancer*.

[147] Chorobik P., Czaplicki D., Ossysek K., Bereta J. (2013). Salmonella and cancer: from pathogens to therapeutics. *Acta Biochimica Polonica*.

[148] Grozdanov L., Raasch C., Schulze J. (2004). Analysis of the genome structure of the nonpathogenic probiotic *Escherichia coli* strain Nissle 1917. *Journal of Bacteriology*.

[149] Grozdanov L., Zähringer U., Blum-Oehler G. (2002). A single nucleotide exchange in the wzy gene is responsible for the semirough O6 lipopolysaccharide phenotype and serum sensitivity of *Escherichia coli* strain Nissle 1917. *Journal of Bacteriology*.

[150] Visvader J. E., Lindeman G. J. (2012). Cancer stem cells: current status and evolving complexities. *Cell Stem Cell*.

[151] Naujokat C., Steinhart R. (2012). Salinomycin as a drug for targeting human cancer stem cells. *Journal of Biomedicine and Biotechnology*.

[152] Ben-Jacob E. (2013). Engineering Trojan-horse bacteria to fight cancer. *Blood*.

[153] Forbes N. S., Coffin R. S., Deng L. (2018). White paper on microbial anti-cancer therapy and prevention. *Journal for ImmunoTherapy of Cancer*.

[154] Min J.-J., Kim H.-J., Park J. H. (2008). Noninvasive real-time imaging of tumors and metastases using tumor-targeting light-emitting *Escherichia coli*. *Molecular Imaging and Biology*.

[155] Jiang S.-N., Phan T. X., Nam T.-K. (2010). Inhibition of tumor growth and metastasis by a combination of *Escherichia coli*—mediated cytolytic therapy and radiotherapy. *Molecular Therapy*.

[156] Nguyen V. H., Kim H.-S., Ha J.-M., Hong Y., Choy H. E., Min J.-J. (2010). Genetically engineered *Salmonella typhimurium* as an imageable therapeutic probe for cancer. *Cancer Research*.

[157] Duong M. T.-Q., Qin Y., You S. H., Min J. J. (2019). Bacteria-cancer interactions: bacteria-based cancer therapy. *Experimental & Molecular Medicine*.

